# Microfluidic Transportation Control of Larval Zebrafish through Optomotor Regulations under a Pressure-Driven Flow

**DOI:** 10.3390/mi10120880

**Published:** 2019-12-14

**Authors:** Bivas Panigrahi, Chia-Yuan Chen

**Affiliations:** Department of Mechanical Engineering, National Cheng Kung University, Tainan 701, Taiwan; n18047068@mail.ncku.edu.tw

**Keywords:** zebrafish larvae, microfluidic devices, hydromechanical response, optomotor response

## Abstract

To perform zebrafish larvae-related experiments within a microfluidic environment, the larvae need to be anesthetized and subsequently transported into respective test sections through mechanical or manual means. However, anesthetization tends to affect larval sensory perceptions, hindering their natural behaviors. Taking into account that juvenile larvae move naturally within their environment by accessing visual as well as hydromechanical cues, this work proposes an experimental framework to transport nonanesthetized larvae within a microfluidic environment by harmonically tuning both of the aforementioned cues. To provide visual cues, computer-animated moving gratings were provided through an in-house-developed control interface that drove the larval optomotor response. In the meantime, to provide hydromechanical cues, the flow rate was tuned using a syringe pump that affected the zebrafish larvae’s lateral line movement. The results obtained (corresponding to different test conditions) suggest that the magnitude of both modalities plays a crucial role in larval transportation and orientation control. For instance, with a flow rate tuning of 0.1 mL/min along with grating parameters of 1 Hz temporal frequency, the average transportation time for larvae that were 5 days postfertilization was recorded at 1.29 ± 0.49 s, which was approximately three times faster than the transportation time required only in the presence of hydromechanical cues.

## 1. Introduction

Zebrafish (*Danio rerio*) are unarguably considered to be one of the most important animal models that have been used for studies on neurobiological development [1], cardiac development [2,3,4], pharmacological research [5], genetic modeling [6], and toxicological research [7,8]. Most zebrafish-related research has been carried out in their early larval stages, where the larvae are transparent and small for applications of powerful imaging tools and genetic modification. In traditional experimental assays, experiments have been performed in a Petri dish under manual control, and the assays have been found to be ineffective in accurately processing a large number of specimens in a short time. To address these issues, several microfluidics modules have been developed in the last two decades that can be used to mount zebrafish embryos to facilitate embryo screening [9,10,11,12,13]. Traditionally, to load and manipulate these embryos within the microfluidic test chambers, they need to be anesthetized and subsequently either forcefully pumped through a pressure-driven flow or manipulated by forceps [9,12]. It can be noted that these early-stage larvae are fragile by nature, and these aforementioned manual processes induce permanent damage to them, leading to their mortality. Moreover, the manual procedure of larvae manipulation requires an operator’s intervention to position the larvae with precision, which is time-consuming by nature and hence hinders high-throughput screening processes for new drug testing. At this point, it can be noted that juvenile zebrafish usually take their environmental cues through multisensory processes that can be affected by several stimulus modalities, such as vestibular inputs, heat, visual stimuli, and hydromechanical cues. However, recent investigations have mostly suggested that both hydromechanical and visual cues play paramount roles in providing sensory inputs to juvenile larvae (regarding coherent information within their environment) [14,15]. Hence, this study hypothesizes that by regulating hydromechanical and visual modalities within the microfluidic platform, juvenile larvae can be manipulated with precision in a noninvasive manner.

Although it is far from fully understanding the complicated behavioral dynamics of zebrafish larvae, the research fraternity widely believes that the lateral line of the zebrafish body mediates the orientation of the body against incoming flow and that the optomotor response (OMR) mediates counterflow [14,15,16,17]. In their natural surroundings, velocity gradients are created through nearby obstructions when the zebrafish larvae encounter a sheared flow. To respond to these external hydromechanical stimuli, a juvenile zebrafish uses a mechanosensory methodology called a lateral line system. The lateral line system runs from head to tail and is comprised of individual sensory organs known as neuromasts [14,15,18,19,20]. These sensory systems are located in canals and are easily accessible on the surface of the developing larvae [20]. The lateral line system provides larvae with the ability to reorient themselves toward the oncoming flow. This behavior is popularly called rheotaxis, and it is essential for larvae in sensing their surroundings so they can survive, detect prey, and swim in a school [15,21]. Due to their rapid development post-hatching, to survive in a hostile environment and to access their food, juvenile zebrafish need to access visual cues, and researchers have realized that zebrafish start to take visual clues 70 h postfertilization. It has been realized that artificial visual stimuli can generate eye movements (optokinetic response (OKR)) as well as body movements (optomotor response (OMR)) [22,23]. A rotational motion, typically in the form of black and white alternative stripes, drive the OKR, whereas a translational motion, typically in the form of stripes projected on a horizontal screen below the larvae, stimulates the OMR. It can be noted that the OKR can be identified when the larvae are three days old, whereas the OMR comes into play only when larvae become four days old. The OMR consists of a set of basic motor components that determines motion direction, where forward swimming and turning are regulated by forward and lateral movements [24]. Juvenile larvae are known to adjust their average swimming speed according to their visual feedback. 

Recent research has demonstrated that zebrafish larvae can be successfully transported to different experimental chambers within the microfluidic platform by regulating their optomotor behavioral response (OMR) [25]. However, for practical purposes, such as a drug delivery system, continuous fluid flow is necessary to maintain a suitable environment as well as to deliver drugs and reagents, etc. Hence, the harmonic regulation of both visual and hydromechanical cues is necessary within a microfluidic environment for the efficient transportation of larvae. In a recent review, the authors specified that optic flow behavior can be integrated with hydromechanical behavior to describe the multisensory behaviors of zebrafish larvae [16]. Very few studies have been conducted from this aspect with conventional experimental assays, suggesting that optic flow modalities dominate the behavioral responses of zebrafish larvae when they are available [14,17]. Still, the collaborative results from previous studies in terms of both cues in a microfluidic environment remain unclear; however, they will be beneficial for animal testing if they can be addressed in a systematic manner. Driven by this motivation and to initialize a new chapter in bioassays, the objective of this study was to design and develop a microfluidic experimental framework through which optical and hydromechanical cues can be regulated for larval manipulation. To provide optical cues, computer-animated gratings were provided from under the larvae, which drove the optomotor response of the larvae, whereas to provide hydromechanical cues, flow rates with different speeds were regulated.

## 2. Materials and Methods

### 2.1. Fish Maintenance and Larvae Preparation

The zebrafish used for this experiment were housed in a commercially available zebrafish core facility (AZ-303, GENDANIO, Taipe Taiwan) with biological and chemical filtration that were in accordance with the set standard protocol [26]. Water quality was maintained at optimal parameters (pH = 6.5–7.5; conductivity: 500–800 μS/cm; ambient temperature: 28 °C), and all the fish were fed twice per day with a mixture of dry flake foods and freshly hatched brine shrimp (*Artemia salina*) according to their ages and sizes. Additionally, the facility was kept at a 14:10 h (light/dark) cycle to maintain a natural circadian rhythm. The zebrafish larvae (4–7 days postfertilization (d.p.f.)) used in this experiment were bred by mating a pair of wild-type adult zebrafish. All experiments were conducted with the approval of the National Cheng Kung University Institutional Animal Care and Use Committee (IACUC). 

### 2.2. Design Details and Experimental Platform

**Figure 1:**
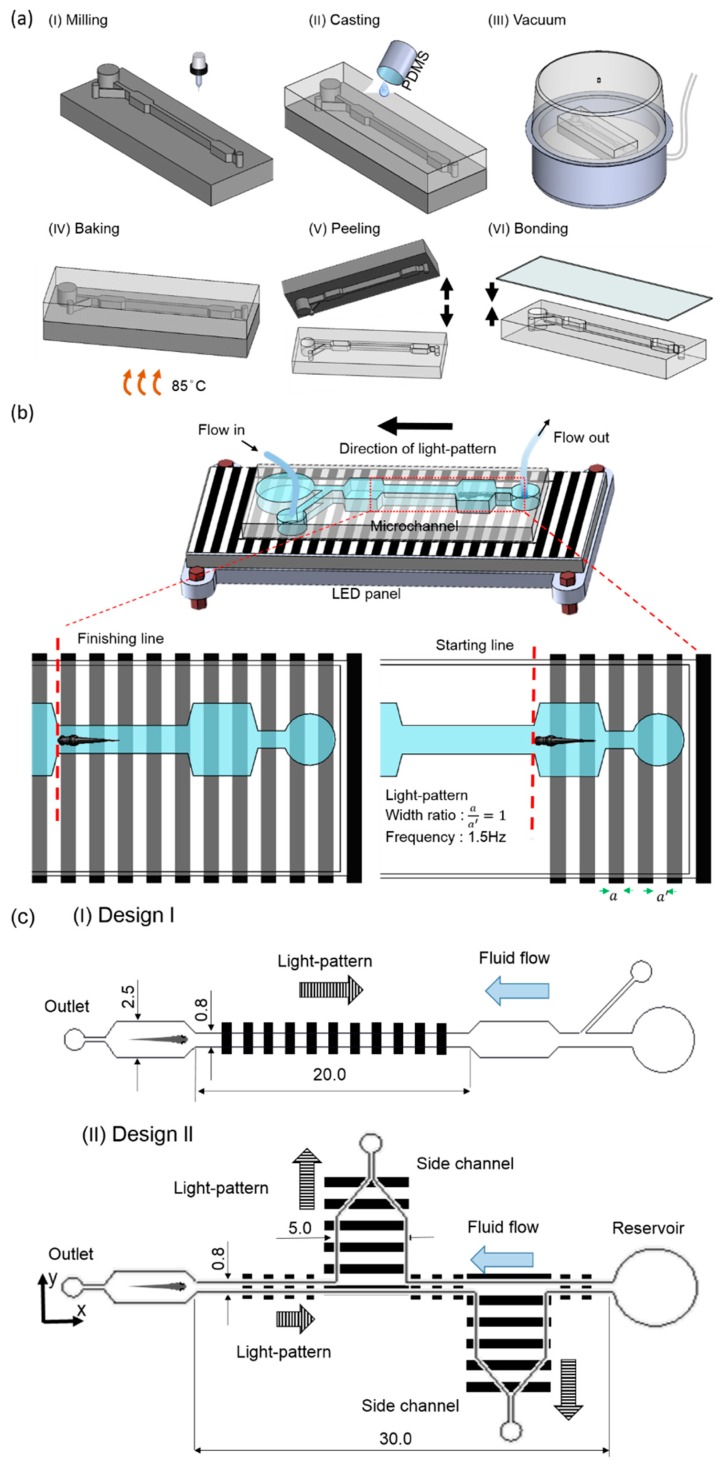
Fabrication and experimental system. (**a** (I–VI)) Fabrication of the microfluidic device consisting of two microwells and one experimental chamber. (**b**) Experimental setup consisting of a microfluidic, flow control, and optical illumination setup platform. The optical setup illuminated the larvae from the bottom with an optimum grating frequency of 1.0 Hz and a grating width ratio ($a/a^{\prime }$) of 1:1. A syringe pump was connected at the outlet to draw fluid, while the fluid was introduced manually at the inlet. The schematics are not to scale. (**c**) Design and dimensional details of both of the microfluidic devices used for the experiments. All dimensions are in mm.

To test the zebrafish larvae migration corresponding to the hydromechanical and optical stimuli within the microfluidic environment, two types of microfluidic devices were fabricated through a series of micromachining and polydimethylsiloxane (PDMS, Sylgard 184, Dow Corning Corp., Midland, MI, USA) casting processes (Figure 1a). Microfluidic device I consisted of two microwells and one experimental chamber, whereas microfluidic device II consisted of two additional test sections for potential drug testing. Side ports were also available to administer drugs and other fluidic requirements. The microchannel layout was first drafted using a commercially available software (Solid Works, Dassault Systèmes SolidWorks Corp., Waltham, MA, USA), and the design was imprinted on an acrylic substrate 5 mm thick using CNC (computerized numerical control) micromachining. To fabricate the microfluidic structure, a mixture of PDMS and curing agent in a ratio of 10:1 were mixed uniformly, degassed in a vacuum chamber, and introduced into the acrylic mold. Subsequently, the whole structure was placed on a hot plate at a temperature of 90 °C for a period of approximately 2 h for the curing process. Subsequently, the PDMS channel was detached from the mold, and an inlet and an outlet were punched through a biopsy puncher. The microchannel was subsequently bonded with another PDMS layer through a plasma cleaning process. It can be noted that polyethylene tubing (0.965-mm outside diameter (OD), 0.58-mm inside diameter (ID)) was only connected at the outlet, and the inlet was open to the environment and acted as a reservoir for the collection of larvae postexperiment.

To generate the OMR response in juvenile zebrafish larvae, moving gratings were generated by illuminating the larvae from their bottoms through an LED panel (Figure 1b). It can be noted that to generate moving gratings, an in-house-developed GUI was used, and the moving gratings were provided along the required direction of zebrafish larvae transportation. The grating frequency and grating width ratio were set as 1 Hz and 1:1, respectively. To provide hydromechanical cues, an infuse/withdraw syringe pump was used, and the flow direction was determined according to the experimental purpose. Zebrafish larvae were placed inside the microwell to test the behaviors of larvae corresponding to the light and hydromechanical stimuli. The movements of zebrafish larvae were recorded using a mobile camera mounted on a tripod holder. Recorded video files (see in the Supplementary Materials, Videos S1 and S2) were replayed, and the larvae’s rostrum was continuously tracked throughout the videos using an open-source video tracking software package such as ImageJ (1.50d, National Institutes of Health, Bethesda, MD, USA) [27]. The design and dimensional details of both microfluidics are illustrated in Figure 1c. It can be noted that the dimensions of both the devices were decided accordingly so that the devices could accommodate zebrafish larvae 4–7 d.p.f., where their length and width typically varied between 0.5 and 0.6 mm and 3.7 and 4.5 mm, respectively [13,28].

### 2.3. Data Analysis

To determine the statistical significance of the different modalities in terms of zebrafish manipulation, both a Student’s *t*-test and an ANOVA test were performed according to the number of variables using the commercially available software SPSS (SPSS, Chicago, IL, USA).

## 3. Results

### 3.1. Behavioral Responses Corresponding to Optical and Hydrodynamic Cues

**Figure 2:**
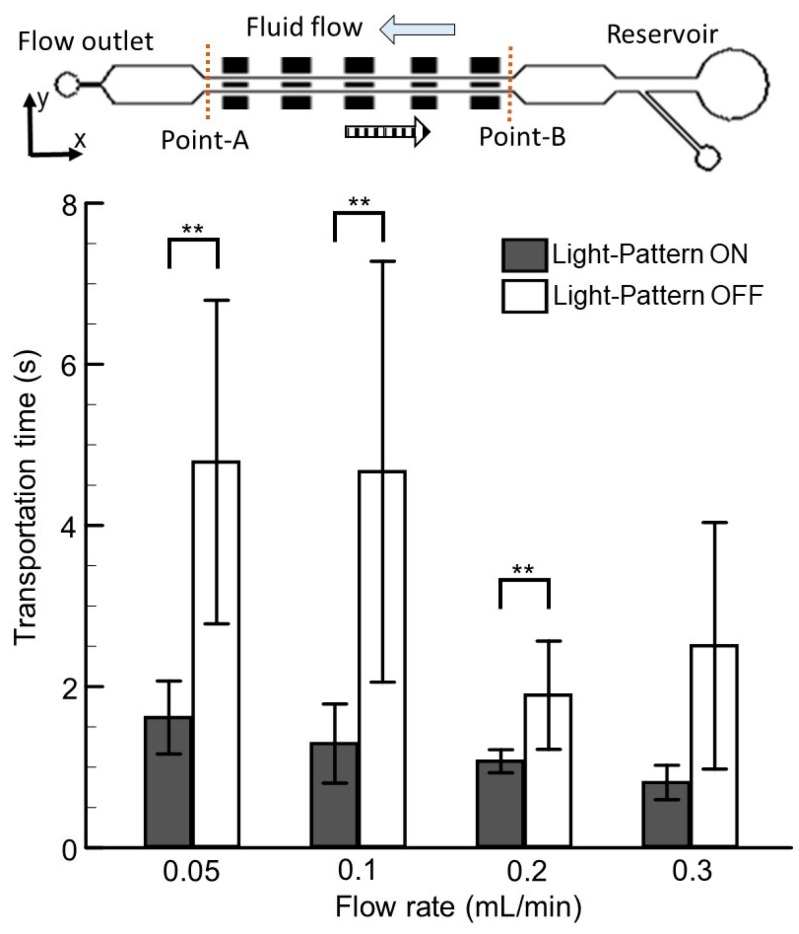
The average transportation time of zebrafish larvae corresponding with the increase in flow rate with the presence and absence of visual cues. Transportation time can be defined as the swimming time taken by the juvenile zebrafish larvae from starting point A to destination point B. Data are illustrated as means ± s.e.m. (n = 10 samples); ** *p*-value < 0.01; paired sample *t*-test.

To test the behavioral responses corresponding to changes in sensory modalities, such as visual and hydromechanical cues, tests were conducted with 5-d.p.f. zebrafish larvae. At this stage of development, the yolk supply runs out, and the juvenile, in search of prey (protozoa), starts using its multisensory modalities [19,29]. As the objective of this work was to transport zebrafish larvae efficiently within the microfluidic environment by regulating only environmental cues, experiments were conducted by positioning the zebrafish larvae in the microchannel and introducing different flow stimuli (0.05–0.3 mL/min) in the presence and absence of optical cues (light pattern ON and OFF). Flow tuning mimicked the test conditions of zebrafish microfluidic assays, where continuous reagent flow was ubiquitous. As the transportation time of zebrafish larvae indicates the effectiveness of swimming larvae (corresponding to the provided stimuli) [22], transportation time was recorded and is illustrated for different test conditions (Figure 2). The data were recorded through measurements of the swimming time of zebrafish larvae from the starting point (point A) to the destination (point B). The transportation time of juvenile larvae was further quantified by averaging the obtained time over several trials (n = 10). To realize the statistical significance of visual cues, a paired sample Student’s *t*-test was performed. It was observed that the swimming time was at a minimum when both the stimuli, i.e., the hydromechanical and optical modalities, were present. For instance, at a relatively low flow rate of 0.1 mL/min, the average swimming time was quantified as 1.29 ± 0.49 s when both the modalities were present compared to an average swimming time of 4.67 ± 2.61 s when the visual cues were absent. A similar trend was observed for other flow rates, including 0.1, 0.2, and 0.3 mL/min, which indicates the importance of both parameters in the microfluidic manipulation of zebrafish larvae. Moreover, the paired *t*-test further depicted that there was a statistically significant difference in the transportation time with the presence and absence of visual cues (paired sample *t*-test, *p*-value < 0.01), indicating the dominance of visual cues over hydromechanical cues even within the microfluidic environment. This agrees well with the literature, where authors have highlighted that within open channels, visual cues dominate larval behavior [14,16,17]. However, it was further observed that with the sole presence of hydromechanical cues, zebrafish larvae could be transported efficiently within the microfluidic environment at relatively higher flow rates of 0.2 and 0.3 mL/min. For instance, at a higher flow rate of 0.2 mL/min, even with the absence of visual cues, the larval transportation time was 1.89 ± 0.67 s, which was marginally higher than the other experimental conditions, where a flow rate of 0.05 mL/min was provided along with the presence of visual cues. 

To further evaluate the success rate of zebrafish transportation corresponding to each modality, a term failing rate was defined. The failing rate can be defined as the percentage of larvae that did not reach their destination (point B) and swam back from their midpath compared to the number of larvae used in this experiment. Still, a higher flow rate with the combination of visual cues proved to be effective in terms of larvae transportation, yet the higher failing rate illustrated the inability of larvae to swim against oncoming fluid currents of higher strength. For instance, it only takes an average minimum of 1.07 ± 0.14 s to transport larvae from a starting point to an endpoint with a flow rate of 0.3 mL/min, yet a failing rate of 20% was recorded even in the presence of optical cues. This inability of zebrafish transportation at the higher flow rate can be described as the inability of juvenile zebrafish to reorient against a higher fluid shear to perform rheotaxis, which is in accordance with recent findings where the authors highlighted that the rheotaxis response diminishes with higher velocities within microfluidic confinement [30]. Still, these explanations do not explicitly provide a convincing concept regarding the diverse behavioral response of larvae in the experimental conditions. Therefore, a follow-up discussion was initiated to emphasize various swimming dynamics in different experimental conditions.

### 3.2. Kinetic Parameters of Swimming Larvae

**Figure 3:**
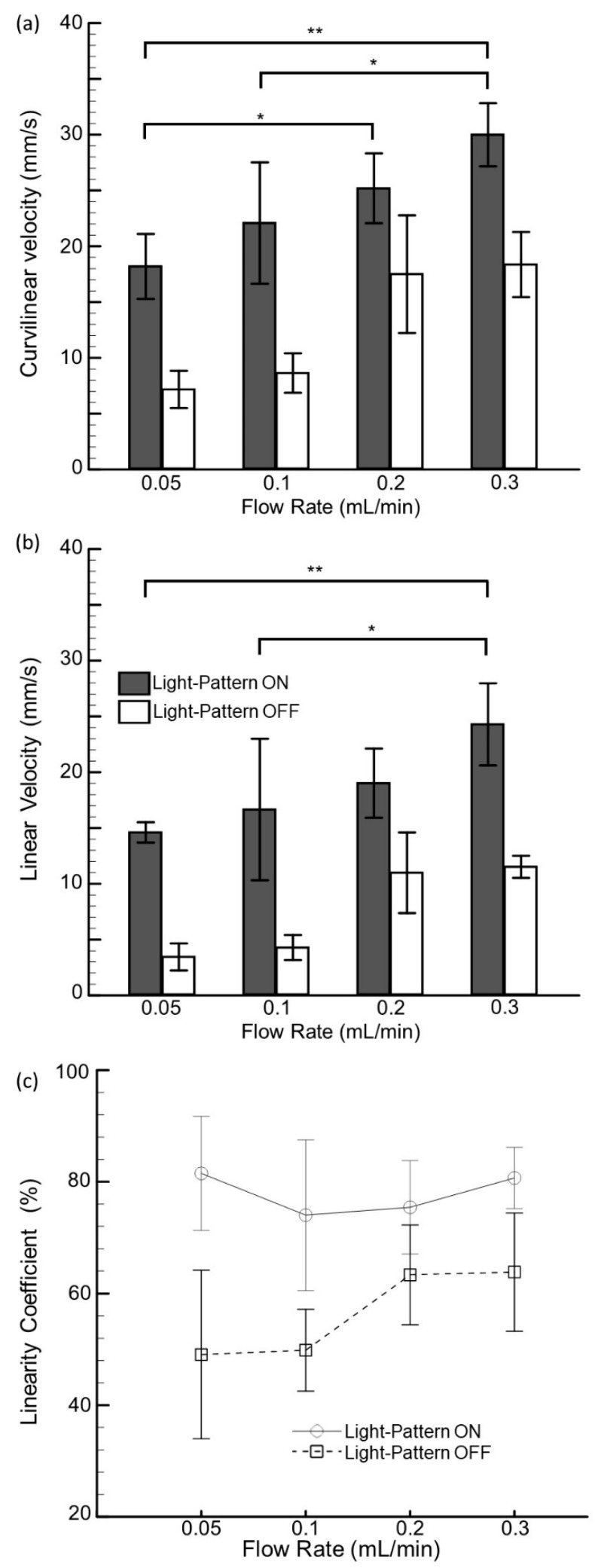
Effects of hydromechanical and visual cues on the swimming kinetic parameters of swimming larvae. (**a**) Curvilinear velocity, which refers to the speed of the actual distance traveled over time. (**b**) Linear velocity, which refers to the speed of the shortest distance traveled over time. (**c**) LIN: curvilinear velocity/linear velocity. Data are illustrated as means ± s.e.m. (n = 10 samples); * *p-*value < 0.05; ** *p-*value < 0.01; ANOVA test.

To delve into the detailed behavioral responses of swimming larvae corresponding to both modalities, we further quantified different swimming parameters, such as curvilinear velocity, linear velocity, and the linearity coefficient, corresponding to different flow rates with the presence and absence of visual cues (Figure 3). The curvilinear velocity is defined as the actual distance traveled by the larvae within the given time period, whereas the linear velocity is defined as the displacement (the shortest path for the zebrafish larvae from the starting point to the endpoint) within changes in the given time period. Further, the linearity coefficient reflects the straightness of the larval path, which was obtained by dividing the curvilinear velocity by the linear velocity. It was observed that with an increase in the flow rate (with or without the presence of visual cues), the swimming velocity of larvae increased significantly, highlighting a relatively shorter transportation time for a higher flow rate. The higher linearity coefficients at higher flow rates (with the availability of visual cues) further depicted that the larvae swam vigorously in a relatively straight line toward their destination. This resulted in a shorter transportation time. Statistical significance (*p*-value < 0.01) was observed (with the presence of visual cues), which corresponded to an increase in the flow rate, further validating the concept of the integration and regulation of both modalities in terms of larvae transportation.

### 3.3. Behavioral Responses Corresponding to Larval Age

**Figure 4:**
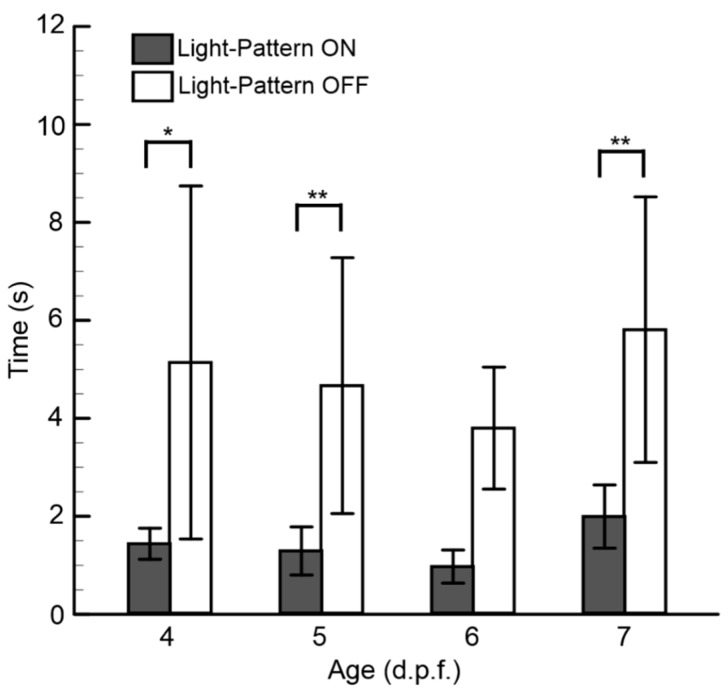
The transportation time of zebrafish larvae of various age groups. Data are illustrated as means ± s.e.m. (n = 10 samples); * *p-*value < 0.05 and ** *p-*value < 0.01 for the paired sample *t*-tests.

To provide quantitative information regarding how both parameters (i.e., the hydromechanical and optical cues) affect the swimming behavior of zebrafish larvae for different age groups (4–7 d.p.f.), experiments (n = 10, zebrafish number) were conducted with the aforementioned microfluidic platform, where a constant flow rate of 0.1 ml/min was imposed while turning the visual cues ON and OFF (Figure 4). It was observed that the swimming time significantly decreased with the presence of visual cues for all groups. With the availability of both modalities, the average swimming time was found to be 0.97 ± 0.33 s for the 6-d.p.f. larvae group and 1.99 ± 0.64 s for the 7-d.p.f. zebrafish larvae group. The differences in transportation time between all groups of zebrafish larvae were negligible with the presence of both modalities. These results indicate that both modalities have essential roles in larvae transportation, and they can be tuned according to age for manipulation within the microfluidic environment. 

### 3.4. The Effects of Visual and Hydromechanical Stimuli on Larvae Transportation in a Microfluidic Network

**Figure 5:**
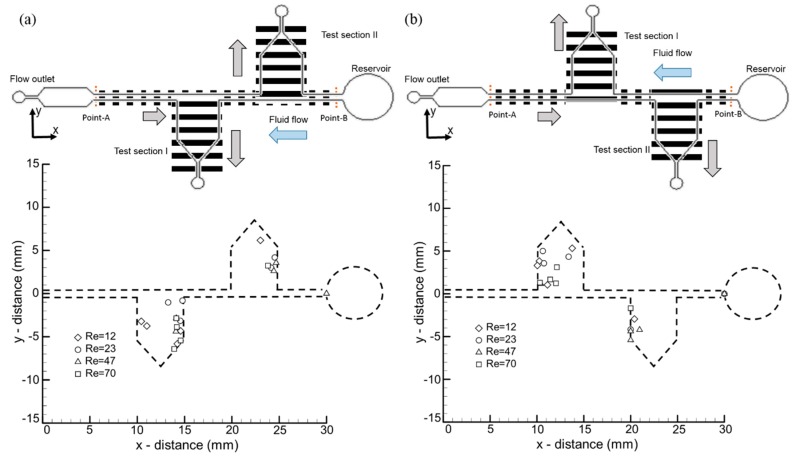
The modified microfluidic channel with two additional test sections, namely test section I and test section II, where the optical stimulus was provided according to the channel opening. The location of the final resident point of the individual zebrafish larvae is depicted in 2D coordinates under different flow conditions. The symmetric and direction-specific nature of larvae migration was tested through a microfluidic device with test section I on the (**a**) right-hand and (**b**) left-hand side of the larvae’s swimming direction.

Although it was evident from the aforementioned results that with the optimal regulation of both visual and hydromechanical cues zebrafish larvae of various age groups could be transported in a straight microchannel, the individual effects of both stimuli on larvae transportation in a complex microfluidic network were not clear. Hence, to test the individual effects of both stimuli within a complex microfluidic network, an experiment was conducted in a microfluidic device with two additional test sections on both sides of the larval swimming direction. The experiment was designed in such a way that close to the side channels (coined test section I and test section II), the OMR pattern and the fluid flow direction were transverse to each other (inserted in Figure 5a). This experimental design mimicked a real-time drug-testing scenario, where the influences of fluid behaviors and time effects should be investigated within a microfluidic network. The visual and hydromechanical cues were regulated accordingly, and the final residence points of zebrafish larvae were recorded and are depicted in Figure 5 with different flow rates. It was observed that irrespective of different flow conditions, most larvae followed the visual cues and turned laterally along the moving gratings toward test section I. As most of the larvae followed the optical cues rather than the hydromechanical cues, it could be deduced that upon availability, optic flow modalities dominate the locomotor behaviors of zebrafish larvae within a microfluidic network. This finding agrees well with earlier findings, where researchers stated a similar locomotor response of zebrafish larvae within conventional assays [14,17]. Additionally, a hypothesis could be drawn at this point that the optomotor response of larvae might be asymmetric and direction-specific. Hence, to verify the aforementioned hypothesis, an additional test was conducted in a microfluidic device with identical dimensional details. One alteration in this new device was the side of test section I. With this change, the side fell on the left-hand side of the larval swimming direction (inserted in Figure 5b). A similar trend in zebrafish migration was observed, where most of the larvae followed the optical modalities and traveled toward the side chamber. This further explained that larval migration corresponding to external optical stimuli is symmetric by nature. These aforementioned findings highlight that by using the proposed experimental framework, zebrafish larvae can be efficiently transported within complex microfluidic networks. With the proposed framework, time-responsive physiological changes of nonanesthetized larvae in terms of the effectiveness of newly developed drugs, the dosage amount, and the timing of dosages can be tested at different test sections within a complex microfluidic network. Moreover, with recent advances in imaging techniques that image neurobehavioral dynamics in freely behaving larvae [31,32], this experimental framework can be further utilized to elucidate a time-responsive neurobiological basis.

## 4. Conclusions

This study demonstrates an important experimental framework where, with a combination of hydromechanical and optical cues, zebrafish larvae can be efficiently transported within microfluidic networks. During the experiment, it was observed that both modalities played crucial roles in larvae transportation within complex microfluidic networks. For instance, with optimal parameter settings, the average transportation time for a 5-d.p.f. zebrafish larva was recorded at 1.29 ± 0.49 s. It was further observed that by regulating both parameters, larvae of various age groups were manipulated with precision within the microfluidic platform. This experimental framework can be used for novel drug testing as well as for behavioral screening.

## Supplementary Materials

The following are available online at https://www.mdpi.com/2072-666X/10/12/880/s1, Video S1: Transportation of zebrafish larvae within Design I, Video S2: Transportation of zebrafish larvae within Design II.
